# Guideline adherence in surgical decisions for T1 colorectal cancer after endoscopic resection: large language models vs clinicians

**DOI:** 10.1097/JS9.0000000000003492

**Published:** 2025-09-10

**Authors:** Liangtang Zeng, Qinxing Cao, Junyuan Deng, Junnan Hu, Minghui Pang, Feng Liu

**Affiliations:** a Digestive Endoscopy Center, Shanghai Tenth People’s Hospital, Tongji University School of Medicine, Shanghai, China; b Department of Geriatric General Surgery, Sichuan Provincial People’s Hospital, School of Medicine, University of Electronic Science and Technology of China, Chengdu, China; c Department of Gastrointestinal Surgery, The First People’s Hospital of Yibin, Yibin, China

**Keywords:** clinicians, endoscopic resection, large language models, surgical decisions, T1 colorectal cancer

## Abstract

**Background:**

Patients with T1 colorectal cancer (CRC) often show poor adherence to guideline-recommended treatment strategies after endoscopic resection. To address this challenge and improve clinical decision-making, this study aims to compare the accuracy of surgical management recommendations between large language models (LLMs) and clinicians.

**Methods:**

This retrospective study enrolled 202 patients with T1 CRC who underwent endoscopic resection at three hospitals. We compared the decision-making accuracy of two representative LLMs (ChatGPT-4o and DeepSeek) with that of 29 clinicians in determining whether additional surgery was required after endoscopic resection of T1 CRC. To optimize the inputs for the LLMs, we applied a prompt-engineering strategy that combined role prompting, in-context learning, and few-shot learning.

**Results:**

In clinical practice, we found that the guideline adherence rate after endoscopic resection for T1 CRC was below 80%. We analyzed the pathology reports of 200 patients with T1 CRC, and the results showed that both ChatGPT-4o and DeepSeek significantly outperformed clinical physicians. Subgroup analyses demonstrated that LLMs outperformed doctors regardless of years of experience or professional background. Additionally, the use of Chinese or English input had no significant impact on the performance of the LLMs.

**Conclusion:**

This study highlights the potential of LLMs to improve guideline adherence in the post-endoscopic resection management of T1 CRC.

**HIGHLIGHTS**

- Large Language Models (LLMs) outperform clinicians in surgical decision-making for T1 colorectal cancer.
- This performance advantage is consistent across clinician seniority levels and specialties.
- LLMs exhibit linguistic robustness and can potentially enhance adherence to clinical guidelines.

## Introduction

Endoscopic resection is an important minimally invasive therapeutic approach for stage T1 colorectal cancers (CRCs). According to the Chinese expert consensus and Japanese Society for Cancer of the Colon and Rectum (JSCCR) guidelines, endoscopic resection is considered curative when specific pathological criteria are met. However, additional surgery is recommended if high-risk pathological features are identified after resection^[1]^. In clinical practice, adherence to these guidelines remains inadequate, with concurrent occurrences of undertreatment and overtreatment. Unnecessary additional surgery might increase patients’ risks without clinical benefit, with associated postoperative mortality rates of 1.5% to 2.0%^[2]^. On the other hand, omitting guideline-recommended additional surgery may allow early cancers to progress, negatively affecting patients’ long-term prognosis.

In recent years, LLMs have shown great potential in medicine, effectively analyzing complex medical data, improving decision-making accuracy, and optimizing patient management. For example, ChatGPT has been evaluated in the field of gastroenterology for its ability to provide management recommendations regarding post-colonoscopy screening and surveillance^[3]^, CRC^[4,5]^, and inflammatory bowel disease^[6]^. Theoretically, combining an LLM with carefully engineered prompts and aligning it with post-endoscopic resection management guidelines for T1 CRC could help clinicians more accurately determine whether additional surgery is necessary. This study aims to evaluate whether this “LLM + guideline” strategy can outperform traditional clinician decision-making when deciding on the need for supplementary surgery in patients with T1 CRC after endoscopic resection. This study adheres to the Transparency in the Reporting of Artificial Intelligence (TITAN) guideline^[7]^.

## Methods

**Figure 1.**
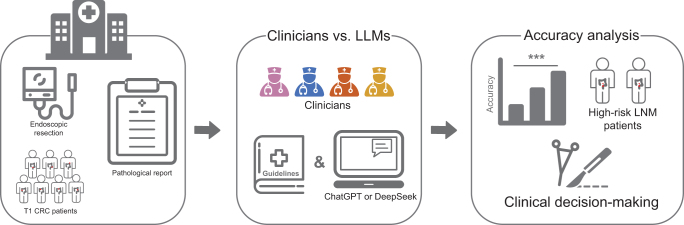
Schematic overview of the study workflow.

We evaluated the accuracy of LLMs in determining the need of additional surgery based on real-world pathology reports of patients diagnosed with T1 CRC after endoscopic resection. Pathology reports were retrospectively collected from 202 patients with T1 CRC who underwent endoscopic resection in three hospitals between 2020 and 2024, representing a wide spectrum of pathology reporting styles. Two cases served as exemplar templates for the LLM prompts, while the remaining 200 cases were used to evaluate the decision accuracy of both the LLMs and clinicians. The study design and workflow are shown in Fig 1. For the LLM assessment, we employed a prompt-based approach that integrating role prompting, in-context learning, and few-shot examples. For the clinician assessment, all pathology reports were converted into a questionnaire and distributed individually to each participating physician for independent evaluation. All case evaluations were completed on 20 May 2025.

Real-world guideline adherence was defined as the proportion of cases in which the care actually delivered conformed to the reference guideline: guideline adherence = (number of guideline-concordant real-world cases)/(total number of cases). Decision accuracy was defined as the proportion of cases in which the treatment plan recommended by the LLM or the clinician aligned with the reference guideline: decision accuracy= (number of guideline-concordant recommendations)/(total number of cases). Further methodological details are provided in Supplemental Digital Content Table S1 and the Supplementary Digital Content 1 (available at: http://links.lww.com/JS9/F95).

## Results

**Figure 2.**
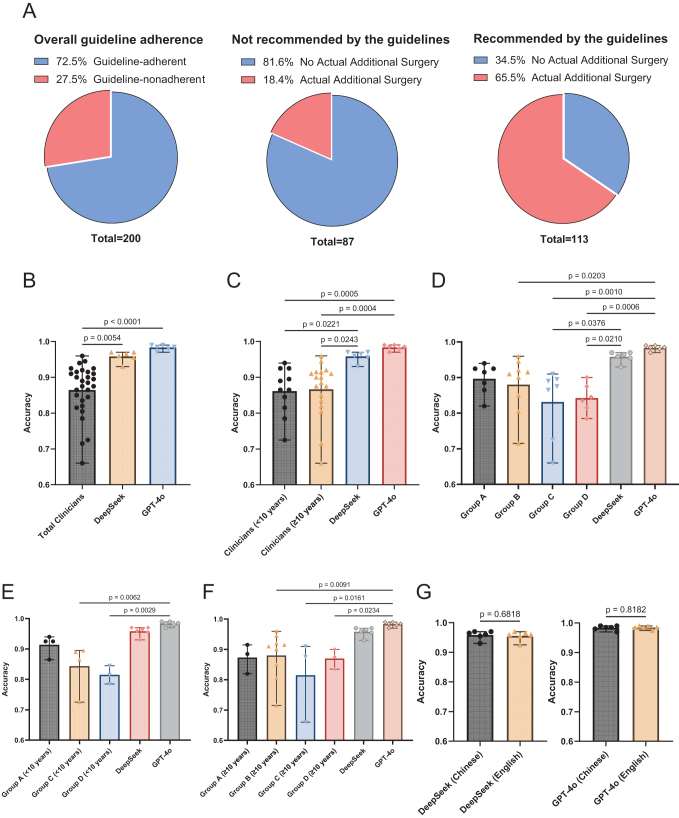
Guideline-adherence rates and performance comparison of LLMs and clinician. (A) Overall guideline adherence in contemporary clinical practice (left), adherence among patients not recommended for surgery (middle), and adherence among patients recommended for surgery (right). (B) Comparison of guideline-adherence performance between LLMs and total clinicians. (C) Comparison of guideline-adherence performance between LLMs and clinicians stratified by clinical experience (<10 years and ≥10 years). (D) Comparison of guideline-adherence performance between LLMs and four clinician groups. (E) Comparison of performance between LLMs and clinicians with <10 years of experience. (F) Comparison of performance between LLMs and clinicians with ≥10 years of experience. (G) Comparison of LLM performance on original Chinese versus translated English input.

Note: Group A, gastrointestinal endoscopists without full mastery of endoscopic resection techniques; Group B, gastrointestinal endoscopists proficient in endoscopic resection techniques; Group C, gastroenterologists not performing endoscopic procedures; Group D, gastrointestinal surgeons. Only statistically significant differences (*P* < 0.05) are shown.

Our findings indicate that in real-world multicenter clinical practice, the overall guideline adherence rate for decisions on additional surgery following endoscopic resection of T1 CRC was 72.5% (Fig. 2A). Specifically, adherence was 81.6% when guidelines suggested no additional surgery, but 65.5% when surgery was recommended (Fig. 2A). We evaluated the accuracy of two LLMs (GPT-4o and DeepSeek-V3) and of clinicians in clinical decision-making. The analysis revealed statistically significant differences among the three groups; post hoc testing demonstrated that both GPT-4o and DeepSeek significantly outperformed the total clinicians group (Fig. 2B), and there was no significant difference between the two LLMs. Further subgroup analysis demonstrated that both models consistently outperformed clinicians, regardless of clinicians’ experience levels (<10 years vs. ≥10 years; Fig 2C).

In subgroup analysis by specialty, both GPT-4o and DeepSeek significantly outperformed clinicians in groups C and D, and GPT-4o was also superior to clinicians in group B (Fig. 2D). Further analysis stratified by specialty and clinical experience showed that GPT-4o significantly outperformed clinicians with <10 years of experience in groups C and D, and clinicians with ≥10 years of experience in groups B, C, and D (Fig. 2E and 2F). We also observed that DeepSeek achieved higher mean accuracy than clinicians in all subgroups, although the differences did not reach statistical significance, possibly due to the small number of clinicians included in each subgroup. These results suggest the potential utility of LLMs in supporting clinical decision-making.

Moreover, we observed considerable inter-clinician variability in decision accuracy within the same subgroup, whereas both GPT-4o and DeepSeek exhibited more consistent performance. Such consistency is important for improving clinical decision-making reliability and supporting effective quality control. Additionally, model performance did not significantly differ between Chinese and English inputs (Fig. 2G), suggesting language differences would not limit the clinical applicability of these LLMs.

## Discussion

To our knowledge, this is the first study using multicenter, real-world pathology reports to evaluate LLM performance in identifying the need for additional surgery after endoscopic resection of T1 CRC. In our study, both GPT-4o and DeepSeek significantly outperformed the clinicians, highlighting the strong potential of LLMs for clinical decision support. Consistent with a recent study, we observed comparable accuracies between GPT-4o and DeepSeek^[8]^. Notably, as an open-source model, DeepSeek offers advantages for rapid integration into clinical practice while meeting data privacy and regulatory standards. Importantly, the multi-institutional dataset in this study encompassed diverse pathology reporting styles, capturing the heterogeneity of real-world clinical practice and thereby strengthening the generalizability and reliability of our findings.

In clinical practice, clinicians’ expertise varies greatly, and decision-making is inherently subjective. Typically, the decision about whether to proceed with additional surgical management after endoscopic resection for patients with T1 CRC rests with the therapeutic endoscopist who performed the resection; in our study, this role was filled by clinicians certified in endoscopic resection techniques – gastroenterologists in Group B and colorectal surgeons in Group D (with ≥10 years of experience). In contrast, advanced LLMs deliver highly consistent decisions and can analyze complex clinical information efficiently and accurately without the need for additional large-scale training. Therefore, integrating LLMs into clinical workflows could improve guideline adherence, reduce decision uncertainty, and ultimately enhance patient outcomes.

This study has several limitations. First, the sample size of 200 cases may not represent the broader patient population. Second, the number of participating clinicians was limited, possibly affecting generalizability. Third, real-world clinical decision-making is often influenced by patients’ personal factors such as refusal of surgery, financial burden, or anxiety, which current LLMs still struggle to handle. Fourth, we focused only on the accuracy of LLM outputs, without analyzing their reasoning processes. Lastly, the applicability of these findings to other settings remains to be confirmed, and further large-scale prospective studies are needed to validate their clinical utility.

In summary, our findings indicate that advanced LLMs achieve exceptionally high accuracy in determining whether patients with T1 CRC require additional surgery following endoscopic resection. These models have the potential to enhance alignment between clinical decision-making and guideline recommendations, and to reduce unnecessary surgical procedures.

## Supplementary Material

## Data Availability

Data supporting the findings of this study are available from the corresponding author upon reasonable request.

## Research registration unique identifying number (UIN)

The study was registered in the Chinese Clinical Trial Registry (ChiCTR) with the Research Registration Unique Identifying Number (UIN): ChiCTR2500104618.
