# Supplementary material for: Guideline adherence in surgical decisions for T1 colorectal cancer after endoscopic resection: large language models vs clinicians
Source: Int J Surg. 2025 Sep 10;112(1):1886–90. doi: 10.1097/JS9.0000000000003492 (PMC12825533; doi:10.1097/JS9.0000000000003492)
Supplement: Supplementary file 1 [file js9-112-1886-001.docx]

**Supplementary Materials**

**Supplementary Table S1.** Prompt for GPT-4 and DeepSeek.

**Supplementary Methods.**

| **Supplementary Table S1. Prompt for GPT-4 and DeepSeek.** |
| --- |
| As a gastroenterologist, please determine whether additional surgery is necessary for this patient with T1 CRC based on the pathological report of endoscopic resection. Please refer to the following criteria for recommending additional surgery.  Criteria for Additional Surgery (additional surgery recommended if any one criterion is met):  1. Positive vertical margin.  2. Poorly differentiation adenocarcinoma, signet-ring cell carcinoma, or mucinous carcinoma  3. Depth of SM invasion ≥ 1000 μm.  4. Positive lymphovascular invasion.  5. Tumor budding (BD2/3).  Few-Shot Learning:  Example 1  (Pathology report from sigmoid colon ESD specimen): High-grade intraepithelial neoplasia with carcinoma (moderately differentiated adenocarcinoma, predominantly located within the mucosal layer, minimal focal infiltration into the submucosal layer <0.5 mm). Tumor budding: BD1. Lymphovascular invasion: negative (-). Vertical and lateral resection margins: negative.  Assessment: Additional surgery is not recommended.  Reason: No risk factors indicating the need for additional surgery were identified in this patient's pathological report.  Example 2  Pathology report  Specimen type: Descending colon ESD resection  Number of lesions: Single  Histopathological diagnosis: Tubular adenoma with moderately differentiated adenocarcinoma  Macroscopic classification: Elevated lesion  Tumor size: Approximately 2.5 cm × 1.5 cm × 1.5 cm  Depth of invasion: Invasion into the submucosal layer (pT1), with depth approximately 1.5 mm  Ulceration: Not present  Lymphovascular invasion: Positive  Perineural invasion: Not identified  Tumor budding: Low-grade  Horizontal and vertical resection margins: Negative  Assessment: Additional surgery is recommended.  Reason: The patient's pathology report shows invasion depth into the submucosal layer (> 1000 µm) accompanied by lymphovascular invasion, both of which are risk factors indicating the need for additional surgery.  Based on the above criteria and examples, please determine whether additional surgery is necessary for this patient according to the provided pathological report. |
| **Example of a response from GPT-4o and DeepSeek**  **Pathology Report Pending Evaluation**  (Sigmoid colon ESD) Moderately differentiated adenocarcinoma with submucosal invasion greater than 1000 μm from the muscularis mucosae. Tumor budding grade: Bd1 (low-grade budding). Tumor cells invade lymphovascular structures, no definite perineural invasion identified. No cancer involvement observed at lateral margins; vertical margin is free of cancer involvement (distance between tumor tissue and vertical margin is approximately 0.2 cm under microscopy). |
| **GPT-4o output**  Assessment: Additional surgery is recommended.  Reason: According to the pathological report, the patient meets two criteria that indicate the need for additional surgery:   1. Depth of submucosal invasion ≥ 1000 μm – Confirmed. 2. Positive lymphovascular invasion – Confirmed.   The other risk factors (poor differentiation, positive vertical margin, high-grade tumor budding) are not present. However, fulfilling any one of the listed criteria is sufficient to recommend additional surgery.  Thus, based on established criteria, additional surgical resection is warranted. |
| **DeepSeek output:**  Assessment: Additional surgery is recommended.  Reason: The patient's pathology report meets two criteria indicating the need for additional surgery:   1. Depth of SM invasion ≥ 1000 μm (explicitly stated as "submucosal invasion greater than 1000 μm"). 2. Positive lymphovascular invasion ("tumor cells invade lymphovascular structures").   While other features (e.g., tumor budding BD1, negative margins, moderately differentiated adenocarcinoma) do not warrant additional surgery, the presence of deep submucosal invasion and lymphovascular invasion confers a significant risk of lymph node metastasis. Thus, completion surgery (typically oncologic resection with lymph node dissection) is advised based on current guidelines. |

**Supplementary Methods.**

This study aimed to evaluate the adherence of LLMs to clinical guidelines in decision-making regarding additional surgery after endoscopic resection of stage T1 CRC and to compare their decisions with clinicians. We tested two currently representative LLMs (ChatGPT-4o and DeepSeek-V3). DeepSeek-V3 is an open-source model that can be conveniently fine-tuned or deployed locally using internal datasets in medical institutions equipped with advanced IT infrastructure ^[1]^. In contrast, ChatGPT-4o is a closed-source hosted model, which is difficult to deploy directly within medical institutions, thus significantly restricting its application in real-world clinical settings ^[1]^. Thus, these two LLMs were selected for evaluation.

The study data were obtained from three hospitals in China. Inclusion criteria were patients undergoing their first endoscopic resection and pathologically diagnosed with T1 CRC. Exclusion criteria included: (1) synchronous or metachronous advanced malignancies; (2) familial adenomatous polyposis, Lynch syndrome, or inflammatory bowel disease (e.g., ulcerative colitis); (3) previous CRC diagnosis; (4) preoperative radiotherapy or chemotherapy; and (5) insufficient data for guideline-based assessment. Ultimately, 202 patients were enrolled. Two cases were randomly chosen as learning examples for the LLM prompt templates, while the remaining 200 cases were formally evaluated. To ensure stability, the LLM analysis was independently repeated six times for all cases.

A total of 29 clinicians participated, categorized into four groups based on their specialties: Group A (gastrointestinal endoscopists without full mastery of endoscopic resection techniques, n=7); Group B (gastrointestinal endoscopists proficient in endoscopic resection techniques, n=9); Group C (gastroenterologists not performing endoscopic procedures, n=7); and Group D (gastrointestinal surgeons, n=6). Each group was further divided into two subgroups based on professional experience (<10 years and ≥10 years), except Group B, which lacked members with less than 10 years of experience. Although, in routine clinical practice, the decision to perform additional surgery after endoscopic resection is made by the therapeutic endoscopist who carried out the procedure—that is, a gastroenterologist or colorectal surgeon with advanced endoscopic resection techniques (Group B and Group D clinicians with ≥ 10 years of experience)—clinicians in the other groups still play important roles throughout the care pathway. Therefore, we compared decision accuracy across all clinician groups.

The prompt templates for the LLMs were collaboratively designed by an author experienced in prompt engineering and a gastroenterologist familiar with CRC guidelines. The prompts included role prompting, in-context learning, and few-shot examples. Specifically, LLMs were instructed, acting as a gastroenterologist, to determine "whether additional surgery is necessary for this patient with T1 CRC based on the pathological report of endoscopic resection." For in-context learning, the model was provided with the relevant recommendations from the JSCCR guidelines on decision-making regarding additional surgical resection after endoscopic treatment of T1 CRC ^[2]^. The JSCCR guidelines were chosen due to their consistency with the Chinese expert consensus. For few-shot examples, two cases were randomly selected from the overall dataset, and their correct expected outcomes were included in the prompt templates to improve the stability of the model's performance on this research task.

The correct responses for all cases were determined jointly by two senior gastroenterologists based on JSCCR guidelines. Any disagreements were resolved through discussion until consensus was reached. The study formally evaluated and recorded the decisions made by ChatGPT-4o, DeepSeek-V3, and all clinicians for each case (a representative example of detailed LLM output is provided in Supplementary Appendix 1). Decision accuracy rates, defined as the proportion of correct cases, were then calculated for each LLM and clinician group. Comparative analyses were performed to assess differences in accuracy between the LLMs and clinician groups.

To assess the effect of input language (original Chinese vs. translated English) on model performance, all Chinese pathological reports were translated into English. Subsequently, a senior gastroenterologist fluent in both languages and experienced in gastrointestinal pathology reviewed and revised these translations to ensure linguistic accuracy and quality.

Statistical analyses and visualizations were performed using GraphPad Prism (version 10.1). Due to potential data non-normality, comparisons between two groups were performed using the Mann–Whitney test, and multiple-group comparisons were conducted using the Kruskal–Wallis test followed by Dunn’s multiple comparisons test with Bonferroni correction. Results are presented as means with ranges. All tests were two-tailed, with *p* < 0.05 considered statistically significant.

**References.**

[1] Sandmann S, Hegselmann S, Fujarski M, Bickmann L, Wild B, Eils R, et al. Benchmark evaluation of DeepSeek large language models in clinical decision-making. Nat Med 2025.

[2] Hashiguchi Y, Muro K, Saito Y, Ito Y, Ajioka Y, Hamaguchi T, et al. Japanese Society for Cancer of the Colon and Rectum (JSCCR) guidelines 2019 for the treatment of colorectal cancer. Int J Clin Oncol 2020;25:1-42.
